# Relationships between parenting style and sibling conflicts: A meta-analysis

**DOI:** 10.3389/fpsyg.2022.936253

**Published:** 2022-08-23

**Authors:** Cong Liu, Mohd Nazri Abdul Rahman

**Affiliations:** Education Faculty, University of Malaya, Kuala Lumpur, Malaysia

**Keywords:** parenting style, sibling conflicts, meta-analysis, moderation effect

## Abstract

Previous studies have shown that sibling conflicts are detrimental to physical and psychological development, as well as long-term human development. Although many studies have discovered relations between parenting style and sibling conflicts, these findings were contentious and did not provide a universal solution. Therefore, the meta-analysis was used as the method to determine the nature and magnitude of the relationships. There were a total of 14,356 participants in the 16 included studies, from which 55 effect sizes were extracted. According to the overall meta-analysis results, authoritative parenting (*r* = –0.201) did have the potential to reduce sibling conflicts. Neglectful (*r* = 0.389), inconsistent (*r* = 0.364), indulgent (*r* = 0.293), and authoritarian (*r* = 0.235) parenting styles were related to sibling conflicts in a positive way. Moreover, the study investigated the effectiveness of several moderator variables, such as children’s gender, age, region, outcome measure, and publication year. The research indicates that authoritative parenting is a protective factor of sibling conflicts. Moderator analyses found that gender, age, region, outcome measure, and publication year played moderating roles in the relationships. Finally, research limitations in the current study and expectations for future research, as well as theoretical contributions and implications for parenting practice, were presented.

**Systematic review registration:**

[https://inplasy.com/inplasy-2022-8-0020/], identifier [INPLASY202280020].

## Introduction

Sibling relationships are vital to individuals because most people’s longest relationships are with their siblings (Kim et al., 2006). Siblings can serve as attachment figures, playmates, protectors, and socializers in emotional support, while they can also be antagonists in conflicts (Davies and Limbo, 2010). Sibling conflict is a common occurrence in families with more than one child (Howe et al., 1997). Studies suggest that sibling conflicts may increase individual anti-social behavior, peer difficulties, sleep issues, and even emotional and behavioral dysfunction within siblings (Bank et al., 2004; Dirks et al., 2015; Breitenstein et al., 2018). According to the family systems theory, sibling conflict involves not only the interaction within the sibling subsystem but also affects interactive patterns of the parental subsystem and the whole family system (Bowen, 1974). Therefore, sibling conflict negatively impacts individual development and sibling relationships, as well as the consistency, cohesion, and balance of the family (Dunn, 2007). Most parents regard sibling conflict as one of the most painful experiences of parenting (Perozynski and Kramer, 1999).

Parenting style, defined as a relatively stable parental behavior pattern and tendency when requesting and providing feedback on their children’s behavior (Baumrind, 1991), has been proved as a predictor of sibling conflict (Milevsky et al., 2011). Researchers expanded Baumrind’s (1971) conceptual model of parenting styles to two specific underlying dimensions: demandingness and responsiveness (Maccoby and Martin, 1983). The two-dimensional framework revealed four parenting styles: authoritative, authoritarian, indulgent, and neglectful (Maccoby and Martin, 1983), as well as a mixed or ambiguous pattern of inconsistent parenting. A study concluded that older siblings tended to feel more hostile to younger siblings because of authoritarian parenting, especially in the second year after the birth of the younger sibling (McHale et al., 2009). While another researcher found that the more authoritarian parenting parents demonstrated in their children, the more harmonious sibling relationships were (Han, 2020). In an indulgent parenting style, researchers discovered that the score of sibling conflicts was high in this parenting style (Sun and Zhang, 2018). However, inconsistent results believed that the indulgent parenting style was negatively correlated with sibling conflicts (Coley et al., 2008). There is also a different sound in the dimension of neglecting parenting style. The neglecting parenting style had the lowest score in positive sibling relationships and the highest score in sibling conflicts of the four parenting styles (Sun and Zhang, 2018), while other studies found that neglecting parenting was associated with fewer sibling conflicts than indulgent parenting (Randjelovic et al., 2021; Yan et al., 2021). Given these research findings, the relationships between parenting style and sibling conflicts remain unclear, suggesting that research gaps need to be filled.

Empirical research may produce varying results because of sampling or other factors (Hunter and Schmidt, 2004). Meta-analysis collects a large number of analysis results from individual empirical cases with the same research topic for statistical analysis, to integrate the research findings and draw a general conclusion (Glass, 1976). Therefore, meta-analysis is required to determine the nature and magnitude of the relationships between parenting style and sibling conflicts. Moreover, the Chinese two-child and three-child fertility policies enacted in 2016 and 2021 encourage couples to have more children to optimize the population structure and ease the aging population (Tatum, 2021). Thus, the number of multi-child families in China has surged. Meanwhile, the significance of sibling relationships has become increasingly prominent in the context of contemporary China (Yan et al., 2021). The obvious evidence is that sibling relationships in the Chinese context have attracted the attention of an increasing number of researchers (Du, 2020; Han, 2020; Yan et al., 2021). Consequently, the current study conducted a multi-language literature search (e.g., Chinese and English) to understand the general status of sibling relationship research in the Chinese and global context and to reduce language bias in the meta-analysis for more comprehensive results (Wang Z. et al., 2021). We conducted a meta-analysis of 16 articles written in English and Chinese to determine the direction and extent of the relationships between parenting styles and sibling conflicts. The findings provide evidence for Chinese and other contexts on how parents reduce their children’s sibling conflicts in family systems by adjusting parenting styles.

## Parenting style

Researchers identified four parenting styles, namely, authoritative, authoritarian, indulgent, and neglectful (Baumrind, 1971; Maccoby and Martin, 1983), as well as a mixed or ambiguous pattern of inconsistent parenting. Authoritative parents who are demanding but also warm, rational, and receptive monitor impart clear standards for their children’s behavior (Baumrind, 1971; Steinberg et al., 1994). Authoritarian parents are highly demanding and controlling, but they are not warm and responsive as they value obedience and expect their commands to be followed without explanation (Boz and Ergeneli, 2014). Indulgent parents, also known as permissive parents, are unconventional, with high responsiveness but low demand and control. They do not require mature behavior from their children and generally avoid conflict in parent–child interactions (Baumrind, 1971). Neglecting parents have low demand/control, as well as responsiveness/acceptance, which is the polar opposite of the authoritative style (Maccoby and Martin, 1983). Finally, there is the inconsistent parenting style, which is not a pure style but may be a combination of several of the above parenting styles, with high and low acceptance and control in parent–child interactions (Dornbusch et al., 1987).

## Sibling conflicts

In the siblings’ dyad, the destructive relationship (sibling conflict) is characterized by frequent, escalating, and intense hostility, as well as high levels of indifference and emotional detachment (Davies et al., 2019). According to the social learning theory (Bandura and Walters, 1977), sibling relationships are essential for children to learn how to interact socially, emotionally, and behaviorally with others (Breitenstein, 2015). For instance, intense hostility and high levels of apathy in sibling conflicts may be learned and applied to parent–child interactions and peer communications. Some previous studies have demonstrated that sibling conflict was a significant predictor of anti-social behavior (Criss and Shaw, 2005), emotional dysfunction (Dirks et al., 2015), and later violent tendencies and poor adjustment (Howe and Recchia, 2006). Therefore, based on numerous research findings, the social learning theory is appropriate for the discussion of sibling conflict, and the adverse effects and relieving strategies of sibling conflicts should also be concerned.

### Relationships between parenting style and sibling conflicts

Most research on the predictors of sibling conflicts has focused on demographic variables, such as sibling gender, age, birth order, family structure, and family socioeconomic status (Criss and Shaw, 2005; Van Volkom et al., 2011). However, studies consistently suggest that the interactive processes of the family are more predictive of sibling relationships than demographic factors (MacKinnon, 1989; Kim et al., 2006). Parenting style is one of the interactive family processes that have received attention in sibling conflict research. Specifically, researchers have concluded that parenting styles may impact sibling conflicts directly or indirectly (Milevsky et al., 2011). In addition, according to the effect mechanism model of parenting styles, parental involvement/coaching, and sibling conflicts, parental involvement/coaching in conflicts plays a major role in the relationships between parenting style and sibling conflicts (McHale et al., 2000). That is, parents’ efforts to settle conflicts between their children may make conflicts worse. Moreover, studies that contend that parents unintentionally stimulate sibling conflicts through social learning (Bandura and Walters, 1977) are consistent with the similarities in conflicts between parent–child and sibling relationships (Bryant and Crockenberg, 1980; Milevsky et al., 2011).

Indulgent parents are less demanding and controllable over their children (Maccoby and Martin, 1983), they may be less involved in and intervene in sibling conflicts. Meanwhile, authoritative and indulgent parents are highly supportive and responsive to their children (Baumrind, 1971), and parents with these two parenting styles are more likely to provide emotional support and response to each child in sibling conflict. Even though authoritative parents are demanding and have clear standards for their children’s behavior, this is not unexplained obedience but rather warm and rational counsel (Steinberg et al., 1994). This has been evidenced by studies, which show that authoritative and indulgent parenting styles were associated with close parent–child relationships (Scharf, 2014; Osorio and González-Cámara, 2016). Through social learning (Bandura and Walters, 1977), closeness in parent–child relationships may also manifest in sibling relationships, resulting in less conflict and more intimacy between siblings. Based on the review presented above, we suggest Hypotheses 1 and 2.

*H1: Authoritative parenting style is negatively associated with sibling conflicts.*

*H2: Indulgent parenting style is negatively associated with sibling conflicts.*

Numerous studies have found that authoritarian and neglectful parenting styles with low response and support positively relate to parent–child conflict (Shek et al., 1998; Aloia and Warren, 2019). According to social learning theory and family system theory (Bowen, 1974; Bandura and Walters, 1977), parent–child conflict affects interactions in sibling subsystems and is likely to present conflict patterns in the subsystem. Furthermore, the authoritarian parenting style emphasizes parental control and demand, and it is likely to intervene in sibling conflicts. Therefore, we suggest Hypotheses 3 and 4:

*H3: Authoritarian parenting style is positively related to sibling conflicts.*

*H4: Neglectful parenting style is positively related to sibling conflicts.*

Inconsistent parenting has been described as using disparate practices across time (Gardner, 1989). Research suggests that inconsistent parenting styles may be linked to children’s conduct problems and negative parent–child relationships. Inconsistent parenting styles are characterized by a lack of rules, a failure to supervise their children, and the use of erratic punishments and rewards (Patterson, 1982). Based on children’s manifestation of problem behavior to interact in the sibling subsystem and parent–child relationship conflict similarity in the sibling subsystem, we propose Hypothesis 5:

*H5: Inconsistent parenting style is positively related to sibling conflicts.*

## Moderators of the relationships between parenting style and sibling conflicts

### The gender of siblings

Gender differences in children have been identified as critical in the emergence of sibling conflict and how children respond to parenting styles (Hosokawa and Katsura, 2019). It is vital to recognize that empathy is a gendered trait, with girls generally being more sensitive to the feelings and emotions of others than boys. That is, girls have higher levels of empathy than boys (Rose and Rudolph, 2006). Researchers discovered that empathy in both firstborns and second-borns was negatively associated with sibling conflicts (Lam et al., 2012). The previous study also confirmed gender differences in sibling conflicts (Snyder et al., 2005). Therefore, boys may be more likely than girls to engage in sibling conflicts. Boys and girls also reacted differently to parenting styles, with competitive boys being more resistant to high-control and demanding parenting styles and sensitive girls being more resistant to low-response and supportive parenting styles (Van Volkom et al., 2011; Bayram, 2014). Thus, the gender of siblings may play a moderating role in the relationship between parenting style and sibling conflicts. In the current study, the percentage of girls was used as a continuous moderator for meta-regression analysis.

### The age of siblings

According to the social comparison theory (Festinger, 1954), people have an inherent desire to evaluate themselves, often compared to others. From the perspective of social comparison theory and family dynamics, differences in relative abilities and interests between siblings diminish over time, creating more opportunities for social comparison and competition (Tesser, 1980; Brody et al., 1994). A longitudinal study also discovered that sibling relationships could become more polarized, comparison and competition could increase, and conflicts could escalate from childhood to early adolescence (Brody et al., 1994). Thus, as a continuous variable, the age of siblings may also play a moderating role in parenting style and sibling conflicts.

### Region

Researchers have shown that many factors affect the parenting style, among which cultural background is essential (Darling and Steinberg, 1993). The idea that the same parenting styles may be interpreted in a variety of ways across cultures (Wang S. et al., 2021) is also supported by the cultural normativeness theory (Lansford et al., 2018). Individualistic societies such as the United States value children’s assertive and independent behaviors, and authoritative parenting has been linked to better outcomes for children (Triandis, 2001; Riany et al., 2021). Whereas in Asian contexts, such as China, with collectivist values, parents tend to be authoritarian and value children’s unobtrusive and obedient behaviors to maintain social harmony (Liu and Guo, 2010; Lansford et al., 2018). This evidence suggests that parenting styles vary across cultures. As a result, the regions of the original studies in this meta-analysis were included as a moderating variable.

### Outcome measure

The meta-analysis included studies that used various scales to assess sibling conflicts. Although these instruments focus on conflicts in negative sibling relationships, some are self-rated by the target sibling, while others are rated by their parents. The various outcome measurement scales may influence the robustness of the research results. Thus, the outcome measure, including SRQ, PEPC-SRQ, KOBS, and others, is investigated as a potential moderator.

### Publication year

Parents’ attitudes, values, and practices are gradually changing as a result of economic globalization and the gradual opening of countries, which may weaken the cultural soil of traditional ideas (Lu et al., 2022). The culture of collectivist and individualistic societies may interact with each other with the development of globalization. For instance, Chinese parents gradually reduce their use of demand and control in parenting, as well as parents in western societies, are beginning to pay attention to the importance of clear standards of conduct for their children (Way et al., 2013). Therefore, era as a continuous variable may play a moderating role in parenting style and sibling conflicts.

## The present study

The current meta-analysis aims to identify the associations between the five parenting styles and sibling conflicts. We explore the strength and direction of the associations between authoritative, authoritarian, indulgent, neglectful, and inconsistent parenting styles with sibling conflicts in Chinese and other contexts. This study hypothesizes that (a) authoritative and indulgent parenting styles are negatively associated with sibling conflicts and (b) authoritarian, neglectful, and inconsistent parenting styles are positively associated with sibling conflicts. In addition, this meta-analysis study tests whether the effect size of the associations depends on demographic information (gender, age, and region) and outcome measure, as well as whether the nature and magnitude of the relationships change over time (publication year).

## Materials and methods

Glass introduced the term meta-analysis in 1976 at the annual meeting of the American Educational Research Association (Shelby and Vaske, 2008). The meta-analysis method gathers many analysis results from individual empirical cases to integrate the research findings and draw a general conclusion (Glass, 1976). This method addresses the shortcomings of traditional empirical research, such as insufficient sample size, low representativeness, and generalizability of research findings (Chen et al., 2015). Therefore, meta-analysis provides a more precise and appropriate research path for investigating the relationships between parenting style and sibling conflicts. The research procedures included (1) searching and collecting studies, (2) coding studies, (3) quantifying heterogeneity and selecting the effect model, (4) analyzing the effect size of included studies, (5) investigating the moderating effects, and (6) analyzing of publication bias.

## Literature search

This study’s literature search procedure strictly adhered to the PRISMA guidelines. The Preferred Reporting Items for Systematic reviews and Meta-Analyses (PRISMA 2020) guideline reflects processes to identify, select, appraise, and synthesize studies. When searching the literature, it is important to present the search strategies for all databases, registers, and websites, along with any filters and limitations that were applied (Page et al., 2021).

The publication time of the included literature was set from the date of database construction to the present. Chinese and English electronic databases were chosen for literature retrieval. The major English literature databases used for the searches were the Web of Science, Scopus, Springer Link, Wiley Online Library, EBSCO, along with Google Scholar. CNKI, VIP, Wanfang, and Longyuan were selected as Chinese literature databases. Parenting style and sibling conflict were entered as two search phrases categories of keywords, while alternative words or synonyms were also included in the search. (1) Parenting style, including parenting, upbringing, parenting mode, rearing style, authoritative, authoritarian, indulgent, neglectful, and inconsistent parenting and (2) sibling conflict, including brother conflict, sister conflict, aggressive sibling, hostile sibling, and negative sibling relationship. The Boolean operator AND was used to link search phrases across categories, and the Boolean operator OR was used to link search phrases within each category. In addition, the references from the collected articles were reviewed as a supplement.

## Criteria for literature selection

The following inclusion criteria were established based on the purpose of this study and the data requirements for calculating effect size using the meta-analysis method. (1) All articles had to be written in Chinese or English, with no published time limit; (2) the studies had to be empirical; (3) the research design was to investigate the relationship between parenting style and sibling conflicts; (4) the studies had to provide information to calculate effect sizes, such as correlation coefficient and sample size; (5) participants were from families with siblings and were from the general population.

## Search results

**FIGURE 1:**
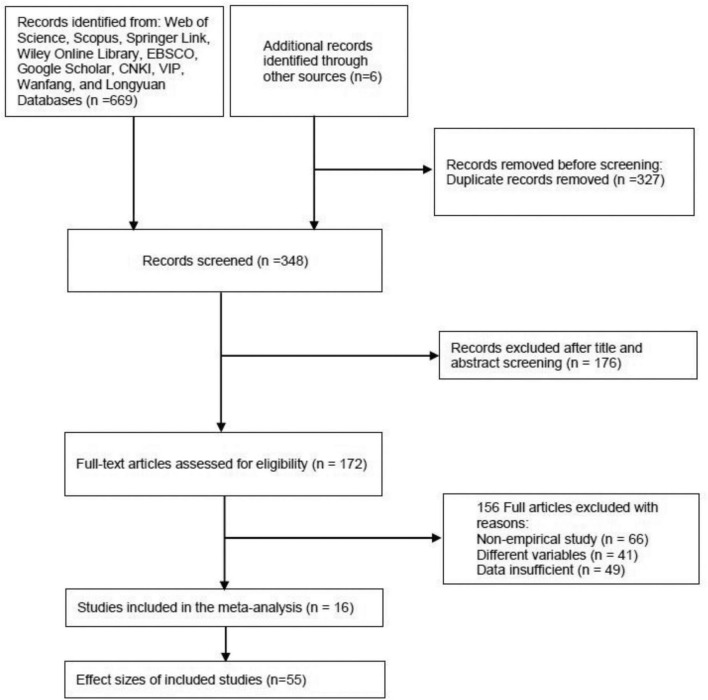
Literature search and inclusion diagram.

Based on the PRISMA flow of article selection, literature identification, screening, eligibility, and inclusion (Page et al., 2021) in this research are shown in Figure 1.

During the first phase of literature identification, we discovered 669 articles published about parenting style and sibling conflict, as well as six articles from other sources. After removing 327 duplicates, 348 records were initially identified and entered the second phase of screening. During the second phase, two researchers determined eligibility by reading the title and abstract of each article. When an article about parenting style and sibling conflict was confirmed as a relevant record, it was retained to be read in full text in the next phase. Following the identification and screening phases, a total of 172 full-text articles were recorded. In the eligibility phase, the recorded articles were further screened. Only empirical studies involving the relationship between parenting style and sibling conflict were retained. The retained articles needed to report sufficient data, such as the sample size and the correlation coefficient. At this stage, a total of 156 articles were excluded.

As a result, this meta-analysis included 16 articles, and these included articles reported 55 independent effect sizes.

### Coding procedure

The study characteristics and relevant statistical data were independently coded by two researchers for the following information: (1) basic information, including author, publication year, outcome measurement, and sample size; (2) participants’ characteristics, such as gender, age, participants’ regions (e.g., Asia, Americas, and Europe), outcome measure (e.g., SRQ, PEPC-SRQ, KOBS, and others), and publication year; (3) parenting styles (e.g., authoritative, authoritarian, indulgent, neglectful, and inconsistent parenting); and (4) effect size (*r*). The coding inconsistencies were resolved through discussion among the two independent coders. The coding results of the included literature are shown in Appendix Table A1.

### Data analysis

The data analyses of this meta-analysis study were conducted by CMA 3.0 (Comprehensive Meta-Analysis Version 3) software. The main analysis contents included: descriptive statistics, publication bias analysis, heterogeneity test and model selection, effect size calculation, and moderator analysis.

#### Descriptive statistics

For each independent sample included in the meta-analysis, descriptive statistics were conducted, including total sample size *N*, independent sample number *k*, minimum and maximum sample size, and minimum and maximum correlation coefficients.

#### Heterogeneity test and model selection

The heterogeneity size information is critical, and only an effective and accurate heterogeneity estimation method can guarantee the estimation accuracy of total effect size in the meta-analysis, and serve as an important reference for model selection and moderator analysis (Chen et al., 2015). The significance of *Q* statistics and *I*^2^ index was used to judge the heterogeneity test, and the test results were used as the basis for model selection.

In the *Q* test method, *p* < 0.10 indicates heterogeneity among independent samples. The *I*^2^ index is the ratio of the inter-group difference to inter-group difference plus intra-group difference. Generally, 25% is taken as the judgment standard. If *I*^2^ < 25%, there was no heterogeneity; if *I*^2^ < 50%, there was moderate heterogeneity; if *I*^2^ < 75%, there was high heterogeneity; and if *I*^2^ ≥ 75%, there was substantial heterogeneity (Higgins et al., 2003). If the results of the analysis revealed heterogeneity, the random-effect model was chosen and subgroup analysis was performed; if there was no heterogeneity, the fixed-effect model was chosen.

#### Effect size calculation

The correlation coefficient *r* was selected to calculate the overall effect size and was converted into *Z*_*r*_ through the *Fisher Z* value for analysis (Peterson and Brown, 2005). Because the sample distribution of the correlation coefficient *r* was skewed, and the variance in the study depended on the correlation, it was necessary to transform *r* into *Z*_*r*_ by *Fisher Z*, and the distribution of *Z*_*r*_ could be approximated to a normal distribution. The calculation formula for *Z*_*r*_ was as follows (Borenstein et al., 2009):


(1) $$Z\,r=0.5*\ln\left(\frac{1+r}{1-r}\right)$$

#### Moderator analysis

When significant heterogeneities in the relationships between parenting styles and sibling conflicts were discovered, moderator analyses were performed to determine which potential moderators could explain these heterogeneities. Mixed-effects between-level *Q* moderator analyses (Borenstein et al., 2010) were used to examine the effects of categorical moderators, including region and outcome measure. Fixed-effect meta-regression (Borenstein et al., 2010) was used to examine the effects of continuous moderators, including gender (the percentage of girls), mean age, and year of publication (Lu et al., 2022).

#### Publication bias analysis

The most common source of errors in the meta-analysis was publication bias, in which positive results were favored by publications (Egger and Smith, 1998). Funnel plots aided in determining the validity of this meta-analysis. Small sample size results would be distributed at the bottom of the funnel plot, while large sample size results would be distributed at the top of the funnel plot. The funnel plot would be similar to a symmetric inverted funnel if there was no bias, whereas the funnel plot was inclined and asymmetric (Egger et al., 1997).

The graphic results of publication bias were also supported by the fail-safe ratio (*N*_*fs*_). *N*_*fs*_ was defined as the number of unpublished studies required to reduce the effect size from significant to insignificant. In general, *N*_*fs*_ was compared to 5*k*+10 (*k* was the number of effect sizes) (Rosenthal, 1979). If *N_*fs*_* > 5k +10, the combined research results are less affected by publication bias (Becker, 2005). Finally, Egger’s regression was used as another method of measuring publication bias in this study.

## Results

### Descriptive statistics

In this meta-analysis, the literature search, identification, screening, eligibility, coding, and extracting process generated 16 included articles. Appendix Table A1 shows the basic information (i.e., coding results) of the included articles. These articles were published between 1997 and 2022, and 10 of them have been published in the last decade. The five included studies were conducted in European countries, three in the Americas, and eight in Asia.

The number of effect values *k*, total sample size *N*, minimum and maximum samples, and correlation coefficients *r* from the analysis of the relationship between the five parenting styles and sibling conflicts in the included articles are shown in Table 1. From the included articles, 55 effect sizes *r* were extracted. We extracted 19 correlation coefficients between authoritative parenting and sibling conflicts and 15 correlation coefficients between authoritarian parenting and sibling conflicts. The study on the relationship between authoritarian parenting and sibling conflicts had the largest sample size of 11,593 participants, while the study on the relationship between inconsistent parenting and sibling conflicts had the smallest sample size of 2,887 participants. When the minimum absolute value and maximum absolute value of the correlation coefficient were compared, the effect sizes differed significantly.

**TABLE 1:** Descriptive statistics of the included studies.

Parenting Style	*k*	Sample		*N*	| *r*|	
		Min	Max		Min	Max
Authoritative	19	71	3,681	11,414	0.100	0.343
Authoritarian	15	32	1,493	11,593	0.047	0.532
Indulgent	9	181	1,365	5,426	0.188	0.455
Neglectful	6	181	1,365	2,968	0.172	0.535
Inconsistent	6	119	1,365	2,887	0.228	0.491

### Heterogeneity test and model selection

The significance of *Q* statistics and the *I*^2^ index were used in this meta-study to determine whether there was heterogeneity among studies under five parenting styles, as well as the basis of the selection random-effect model or fixed-effect model. Table 2 shows the results of the heterogeneity test.

**TABLE 2:** Heterogeneity test results.

Parenting Style	*N*	Heterogeneity			Effect model
		*Q*	*p*	*I* ^2^	
Authoritative	11414	129.050	0.000	86.052	Radom-effect
Authoritarian	11593	333.967	0.000	95.808	Radom-effect
Indulgent	5426	246.772	0.000	96.785	Radom-effect
Neglectful	2968	54.118	0.000	90.761	Radom-effect
Inconsistent	2887	27.294	0.000	81.681	Radom-effect

*Q* test of the five parenting styles showed *p* < 0.05, indicating heterogeneity between studies. Furthermore, all *I*^2^ values greater than 75% indicated a high degree of heterogeneity between studies (Higgins et al., 2003). Therefore, the random-effect model was used to calculate the combined effect size of the five parenting styles and sibling conflicts.

### Overall effect sizes

The results of combined effect sizes and 95% confidence intervals are shown in Table 3. All combined effect sizes were statistically significant (*p* < 0.05). The standard of effect size suggested by Cohen was adopted to judge the size of the combined effect, which was 0.1 ≤ *r* < 0.3 small effect; 0.3 ≤ *r* < 0.5 intermediate effect; *r* ≥ 0.5 strong effect (Cohen, 1988). There was a small and negative effect between authoritative and sibling conflicts, and a small and positive effect between authoritarian and indulgent parenting styles and sibling conflicts, with the combined effect size of *r* = –0.201, *r* = 0.235, and *r* = 0.293, respectively. The other two parenting styles had an intermediate and positive effect on sibling conflicts. The analysis results support the hypotheses of H1, H3, H4, and H5 but do not support the hypothesis of H2.

**TABLE 3:** Overall effect size results and publication bias tests.

Parenting Style	*k*	*N*	*r*	95%CI	Z	*N*_*fs*_	Egger’s *p* (2-tailed)
Authoritative	19	11414	–0.201	–0.256 to –0.146	–6.961***	1556	0.445
Authoritarian	15	11593	0.235	0.124–0.341	4.087***	1015	0.221
Indulgent	9	5426	0.293	0.148–0.426	3.863***	894	0.616
Neglectful	6	2968	0.389	0.273–0.494	6.149***	636	0.879
Inconsistent	6	2887	0.364	0.277–0.445	7.691***	552	0.691

***p < 0.001.

### The effect sizes of moderator variables

Heterogeneity test results showed that there was a high degree of heterogeneity in all the included studies on the relationship between the five parenting styles and sibling conflicts, which could be influenced by moderating variables. In this study, sample characteristics such as gender, age, region, and other variables such as outcome measure and publication language of included articles were considered as moderating variables for moderating analysis, so as to investigate whether these factors of research have a moderating effect on heterogeneity.

#### Categorical variables

Table 4 shows the results of between-level *Q* moderator analyses for categorical moderators (e.g., region and outcome measure).

**TABLE 4:** Moderating effect of categorical variables.

Parenting Style	*Q*_*B*_	Moderator	*k*	N	*r*	95% CI	Z
Authoritative	24.443***	Asia	9	6,628	–0.227	–0.250 to –0.166	–6.207***
		Americas	1	130	0.230	0.060–0.387	2.639**
		Europe	9	4,656	–0.281	–0.442 to –0.102	–7.321***
Authoritarian	9.490**	Asia	8	7,130	0.260	0.104–0.404	3.216***
		Americas	3	263	0.314	0.199–0.420	5.172***
		Europe	4	4,200	0.107	0.027–0.186	2.608**
Inconsistent	2.573*	Asia	4	2,568	0.393	0.391–0.486	7.035***
		Europe	2	319	0.278	0.156–0.391	4.369***
Authoritative	28.718*	SRQ	8	3,598	–0.299	–0.334 to –0.152	–5.675***
		PEPC-SRQ	3	960	–0.271	–0.329 to –0.211	–8.564***
		KOBS	2	400	–0.177	–0.270 to –0.079	–3.541***
		OTHER	6	6,456	–0.268	–0.325 to –0.209	–8.615***
Authoritarian	98.425***	SRQ	6	3,447	0.349	0.273–0.421	8.446***
		PEPC-SRQ	3	960	0.346	0.130–0.532	3.067*
		KOBS	2	400	0.083	–0.015 to 0.180	1.653
		OTHER	4	6,786	0.130	–0.085 to 0.334	1.184*
Neglectful	21.534***	SRQ	2	1,738	0.444	0.406–0.481	19.881***
		PEPC-SRQ	1	649	0.245	0.180–0.308	7.185***
		KOBS	3	581	0.350	0.261–0.434	7.263***
Inconsistent	9.066*	SRQ	3	1,857	0.446	0.369–0.516	10.250***
		PEPC-SRQ	2	830	0.323	0.170–0.460	4.029***
		OTHER	1	200	0.228	0.092–0.355	3.257**

*p < 0.05, **p < 0.01, ***p < 0.001.

The participants’ regions (Asia, the Americas, and Europe) could significantly moderate the relationships between authoritative, authoritarian, and inconsistent parenting styles and sibling conflicts. The disparities between the three continents were enormous for the authoritative parenting style. In America (*r* = 0.230), there was a positive correlation between authoritative parenting style and sibling conflicts, whereas, in Asia (*r* = –0.227) and Europe (*r* = –0.281), there was a negative correlation. Moreover, the strongest correlations between authoritarian parenting and sibling conflicts were found in the Americas (*r* = 0.314), with Asia (*r* = 0.260) having the second highest correlation and Europe (*r* = 0.107) having the weakest. For inconsistent parenting, only Asian and European moderating analyses were performed due to the absence of a sample from the Americas. The results showed that the relationship between inconsistent parenting styles and sibling conflict was stronger at a medium level in Asia (*r* = 0.393) and with a small effect size in Europe (*r* = 0.278).

The outcome measure (SRQ, PEPC-SRQ, KOBS, and OTHER) could significantly moderate the relationships between authoritative, authoritarian, neglectful and inconsistent parenting styles, and sibling conflicts. Compared to other measuring tools, the SRQ scale had the strongest correlation in relationships between authoritative (*r* = –0.299), authoritarian (*r* = 0.349), neglectful (*r* = 0.444), and inconsistent (*r* = 0.446) parenting styles and sibling conflicts.

#### Continuous variables

The meta-regression results of continuous moderators are shown in Table 5.

**TABLE 5:** Moderating effects of continuous variables.

Moderator	k	Estimate	Standard error	95%CI	Z
**Authoritative**					
Publication year	17	0.005	0.001	0.003– 0.007	5.29***
**Authoritarian**					
Mean age	14	–0.030	0.003	–0.035 to –0.025	–11.42***
**Indulgent**					
Mean age	8	0.018	0.003	0.012– 0.024	6.31***
**Neglectful**					
Publication year	6	0.021	0.013	0.006– 0.039	9.69***
% Girls	6	0.065	0.021	0.022–0.097	4.87***
**Inconsistent**					
% Girls	6	0.056	0.016	0.024–0.088	3.43***

***p < 0.001.

Gender (the proportion of girls) significantly moderated the relationship between neglectful and inconsistent parenting styles and sibling conflicts. The proportion of girls could positively predict the effect sizes of the relationships between the two parenting styles and sibling conflicts, indicating that the greater the proportion of female siblings in the samples, the stronger the positive effects of the relationships between neglectful and inconsistent parenting styles and sibling conflicts.

The participants’ average age significantly moderated the relationship between authoritarian and indulgent parenting styles and sibling conflicts. The older age of target children could positively predict the effect sizes of indulgent parenting and negatively predict the effect sizes of authoritarian parenting styles.

The publication year may moderate the relationship between authoritative and neglectful parenting styles and sibling conflicts in a positive and significant way. Specifically, as time goes on, the authoritative parenting style might have a greater negative prediction, while neglectful parenting might have a greater positive prediction of sibling conflicts.

### Publication bias analysis

**FIGURE 2:**
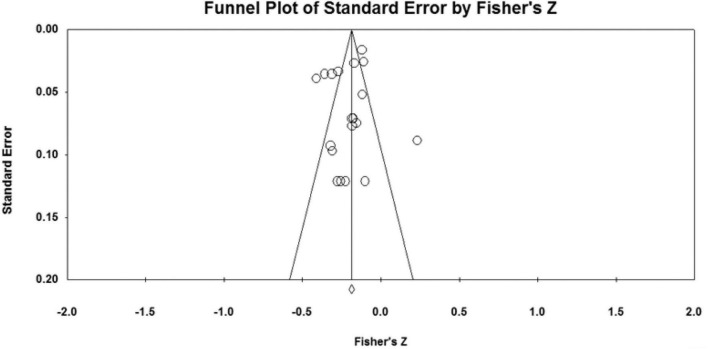
Funnel plot of the relationship between authoritative parenting and sibling conflicts studies.

**FIGURE 3:**
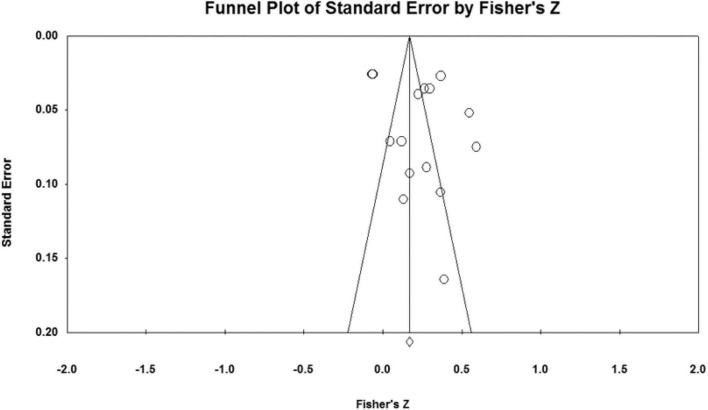
Funnel plot of the relationship between authoritarian parenting and sibling conflicts studies.

**FIGURE 4:**
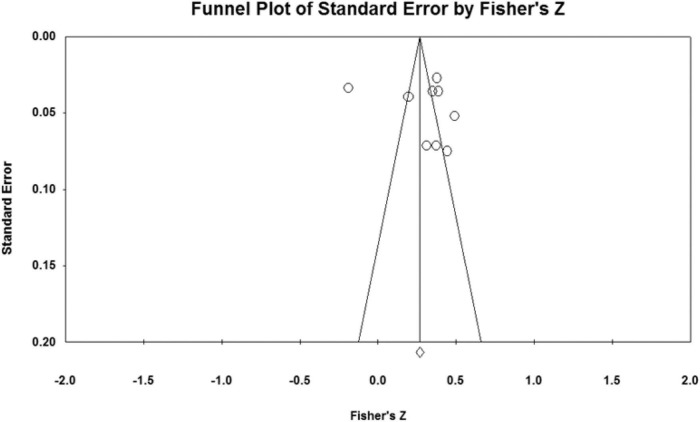
Funnel plot of the relationship between indulgent parenting and sibling conflicts studies.

**FIGURE 5:**
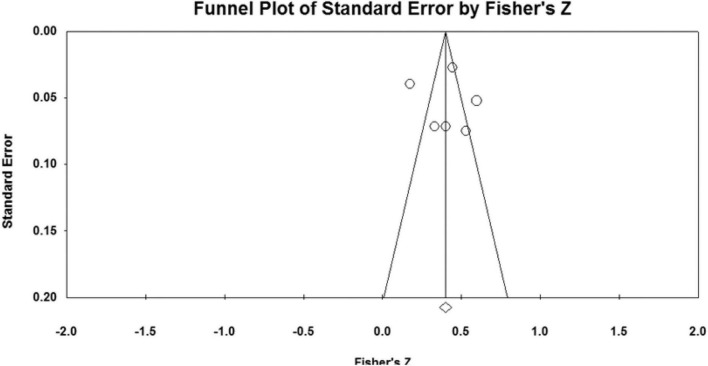
Funnel plot of the relationship between neglectful parenting and sibling conflicts studies.

**FIGURE 6:**
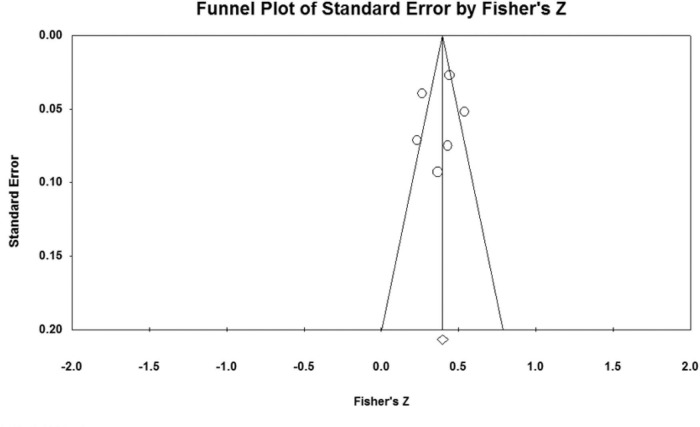
Funnel plot of the relationship between inconsistent parenting and sibling conflicts studies.

The effect sizes of the relationships between five parenting styles and sibling conflicts examined in the study were all concentrated at the top of the symmetrical funnel plots. The funnel plots are shown in Figures 2–6. The combined effect sizes were located in the symmetrical midline of the funnel plots, and all studies were evenly distributed on both sides and were centered at the top of the funnel plots. The funnel plots demonstrated that there was no risk of publication bias. Considering the subjectivity of the graphic results, this study combined the fail-safe ratio *N*_*fs*_ and Egger’s linear regression to support the previously stated the conclusion of publication bias.

The results of the *N*_*fs*_ test and Egger’s regression test are shown in Table 3. The fail-safe ratio revealed that a total of 1,556 studies with invalid results were required to invalidate the effect size for authoritative parenting style, 1,015 studies for authoritarian parenting, 894 studies for indulgent parenting style, 636 studies for neglecting parenting style, and a total of 552 invalid results studies were required to invalidate the effect size for inconsistent parenting style. These *N*_*fs*_ values were far greater than the values of 5*k* +10, indicating that the existing conclusions were not easily overturned, that there was no publication bias in the study, and that the conclusions were valid. In addition, Egger’s linear regression intercept was not significant (*p* > 0.050), which also proved that there was no publication bias in the study.

## Discussion

The meta-analysis method was used in this study to conduct a quantitative and integrated analysis of 16 included articles on the relationships between parenting style and sibling conflicts. The total sample size was 14,356 and 55 effect sizes *r* were extracted from the included articles. Because of the high heterogeneity in this study, random-effect models were used to calculate all of the combined effect sizes. The combined effects of authoritative, authoritarian, indulgent, neglecting, and inconsistent parenting styles on sibling conflicts were calculated, respectively. Subgroup analyses using gender, mean age, region, outcome measure, and publication year as moderators explained the majority of the heterogeneity. Finally, the funnel plot, fail-safe ratio *N* test, and Egger’s linear regression were used to test the publication bias of this meta-analysis. The findings revealed that there was no publication bias, and the findings were stable and reliable.

### The combined effects of the relationship between parenting style and sibling conflicts

The current meta-analysis found a small negative association between authoritative parenting and sibling conflicts, small positive relationships between authoritarian and indulgent parenting styles and sibling conflicts, and medium positive associations between neglectful and inconsistent parenting styles and sibling conflicts. These findings are consistent with some previous research both in Chinese and other contexts (Kim et al., 2006; Sun and Zhang, 2018; Yan et al., 2021). These findings are also consistent with the hypotheses of H1, H3, H4, and H5 proposed according to the social learning theory (Bandura and Walters, 1977) and the family systems theory (Bowen, 1974). Although the positive relationship between indulgent parenting and sibling conflicts does not support hypothesis H2, it is consistent with the findings of previous research (Mohammadi and Zarafshan, 2014; Yan et al., 2021).

### The moderator analyses

In the current study, gender, age, and region of children, outcome measure, and publication year were used as moderating variables for subgroup analysis to explain heterogeneity in the calculation of the combined effects of five parenting styles and sibling conflicts.

There are stronger positive associations between neglectful and inconsistent parenting styles and sibling conflicts when there are more girls among the participants. The results support the prior research finding that empathy and sensitivity are significant gender traits. In general, girls have a higher level of empathy and sensitivity than boys (Rose and Rudolph, 2006). Therefore, the sensitive characteristics of girls require more support and response from parents (Rose and Rudolph, 2006). Girls’ sensitivities are also more likely to perceive inconsistencies in parental support and responsiveness levels across time and context (Bayram, 2014). In addition, parenting styles with low or inconsistent support and response (e.g., neglectful, and inconsistent parenting styles) were negatively associated with empathy in girls (Heynen et al., 2021). Empathy is also a significant gender characteristic that negatively predicts sibling conflict (Rose and Rudolph, 2006; Lam et al., 2012). Specifically, parents of girls who have siblings should consider parenting with high levels of support and responsiveness, and they should be wary of neglectful and inconsistent parenting.

The average age of children had a moderating effect on the relationships between authoritarian and indulgent parenting styles and sibling conflicts. We find that the increase in sibling age promotes indulgent parenting and sibling conflicts. The result is in line with the social comparison theory (Festinger, 1954) we previously mentioned. As siblings age, the differences in their relative skill levels gradually narrow, opening up more opportunities for comparison and competition, even conflict (Tesser, 1980). Especially with indulgent parenting, this parent may be openly affectionate but with few or no limitations. Indulgent parents place few demands on their children’s maturity or performance, and their children hardly struggle to control impulses, such as conflict with others and immaturity (Aghi and Bhatia, 2014). We also find that increasing sibling age can be a protective factor for the association between authoritarian parenting and sibling conflict. This result may be inconsistent with the conclusions of some previous studies (Brody et al., 1994; Kramer et al., 1999). The result could be related to the fact that half of the studies included in this meta-analysis were conducted in Asia. In collectivism, such as in Asian society, parents tend to use authoritarian parenting by enforcing rigid rules, demanding obedience, and employing strategies to force a child to conform (Liu and Guo, 2010). Children in such a cultural context with authoritarian parenting may be well-behaved, polite, and restrained and have fewer sibling conflicts as they grow older (Aghi and Bhatia, 2014; Lansford et al., 2018).

Subgroups were set to Asia, America, and Europe, depending on the region of the participants. The region of participants had a moderating effect on the relationships between authoritative, authoritarian, and inconsistent parenting styles and sibling conflicts. In America, there was a positive correlation between authoritative parenting style and sibling conflicts, whereas in Asia and Europe. Although authoritative parenting was associated with more sibling conflicts in the Americas, only one study was included, so the conclusion was not particularly reliable. In terms of combined effect size, authoritative parenting was still a protective factor for sibling conflicts in Asia and Europe. This finding is partly inconsistent with past research, suggesting that direct instructions given by authoritarian parents in collectivist societies predict children’s obedience, unobtrusive behaviors, and less conflict to maintain social harmony (Liu and Guo, 2010). That is to say, in the current Asian society with economic globalization and social openness, traditional authoritarian parenting in Asia is also influenced to some extent by the democratic and warm response of the authoritative upbringing often used in western culture (Yan et al., 2021). Recently, authoritative parenting styles in Asian societies also began to predict empathy, prosocial behavior, and sibling closeness (Yan et al., 2021; Zhou et al., 2022). In addition, Asian parents need to be wary of inconsistent parenting, which had a medium and positive effect on sibling conflicts. This may be because inconsistent parenting is at odds with the explicit, direct, and consistent instructions and rules that parents have traditionally adopted in Asian societies (Liu and Guo, 2010; Aghi and Bhatia, 2014).

Evaluation tools significantly moderate outcomes in the relationships between authoritative, authoritarian, neglectful, and inconsistent parenting and sibling conflicts. The effect sizes in past studies that adopted children’s self-evaluation (SRQ) are larger than those that used parental evaluation (PEPC-SRQ and others) or adults’ self-evaluation (KOBS) in the relationships between these four parenting styles and sibling conflicts. The finding implies that an independent study may overestimate the effect size due to the common method bias caused by self-evaluation. However, the impact of self-evaluation on meta-analysis is relatively acceptable (Lu et al., 2022) because the meta-analysis collects a large number of results from studies and integrates them to reach a general conclusion (Glass, 1976).

Publication year played a moderating role in relationships between authoritative and neglectful parenting styles and sibling conflicts. The negative effect of authoritative parenting and the positive effect of neglectful parenting on sibling conflict increased over time. The most significant distinction between the two parenting styles is in the dimensions of parental acceptance and response, with authoritative parenting scoring high and neglectful parenting scoring low (Maccoby and Martin, 1983). The findings are consistent with many recent studies (Breitenstein, 2015; Lansford et al., 2018; Yan et al., 2021; Zhou et al., 2022) and family systems theory (Bowen, 1974). In detail, in recent years, with economic globalization and social openness whether in a collectivist or individualist society, warm parental support, and response in parent–child interaction not only promotes the development of a positive parent–child subsystem but also benefits siblings and parental subsystems, as well as the whole family system.

## Limitations and implications

### Limitations

The following were some of the study’s limitations: (1) moderating variables were limited in the meta-analysis. The influencing factors included in this study were not fully covered due to a lack of quantitative analysis of variables. Once sufficient information has been reported about other moderating variables, such as birth order, age difference, gender combination, and socioeconomic status, in the original articles, a future study can investigate these more detailed moderating effects; (2) the number of included articles was limited. The sample size of some moderating variables subgroup analysis was small due to the limited original literature, which might affect the validity of subgroup analysis results to some extent, so that the explanatory role of moderating variables cannot be strongly proven. More cross-institutional and cross-national researchers could be contacted in future to include more original literature in different publication languages to conduct a meta-analysis of relationships between parenting style and sibling conflicts, thereby improving the research’s stability and validity.

### Implications

The findings in current research on the relationships between parenting style and sibling conflicts are consistent with the hypotheses mentioned according to the social learning theory (Bandura and Walters, 1977) and family systems theory (Bowen, 1974), proving the value and importance of these two theories in Chinese and global contexts. Furthermore, the moderating effect analyses on participants’ characteristics (e.g., gender, age, region) verified the gendered trait of empathy (Rose and Rudolph, 2006), social comparison theory (Festinger, 1954), and cultural normativeness theory (Lansford et al., 2018).

For parental practices, authoritarian, indulgent, neglectful, and inconsistent parenting styles positively correlate with sibling conflict. Only the authoritative parenting style has high support and response and less control and demands, producing positive parent–child interaction, which is similarly and positively conducted in sibling subsystem interaction. In addition, parents of girls should be more vigilant about neglectful and inconsistent parenting styles because sensitive and empathetic gender traits make girls sensitive to parental neglect and inconsistent treatment, which may negatively affect their empathy and thus positively predict sibling conflicts. Finally, as children grow, the gaps in their siblings’ relative competence gradually close, providing more opportunities for comparison and competition, even conflict (Tesser, 1980). Indulgent parents only provide affection and lack constraints, and their children will find it difficult to control their behavior as time passes, resulting in immature behavior and conflict in their sibling comparison and competition.

## Data availability statement

The original contributions presented in the study are included in the article/supplementary material, further inquiries can be directed to the corresponding author.

## Author contributions

CL: methodology, software, formal analysis, resources, data curation, and writing—original draft preparation. MR: validation and supervision. Both authors: conceptualization, writing—review and editing, read and agreed to the published version of the manuscript.

## Appendix A

**TABLE A1:** The included articles.

Author	*N*	Title	Journal	% Girls	Mean age	Region	Measure
Randjelovic et al. (2021)	200	Parental educational styles as predictors of perfectionism and quality of sibling relationships among students	Psychological Applications and Trends	50%	21	Europe	KOBS
Yu and Gamble (2008)	130	Pathways of Influence: Marital Relationships and Their Association with Parenting Styles and Sibling Relationship Quality	Journal of Child and Family Studies volume	53%	4.6	America	PEPC-SRQ
Yan et al. (2021)	373	The influence of parenting style on sibling relations among children aged 4–6 in rural areas in Northern China–a regression model	European Early Childhood Education Research Journal	69.44%	5.39	Asia	SRQ
Casey (1997)	93	The relationship between parental intervention into sibling conflict and the quality of children’s sibling relationships	Mater Degree Thesis of California State University	43%	10.3	America	SRQ
Bayram (2014)	172	What determines the siblings conflict resolution strategies of adolescents? Parents, siblings, or temperament?	Master Degree Thesis of Middle East Technical University	100%	15.03	Europe	RCR
Brody et al. (1994)	142	Contributions of Family Relationships and Child Temperaments to Longitudinal Variations in Sibling Relationship Quality and Sibling Relationship Styles	Journal of Family Psychology	45.07%	8	Europe	SRQ
Meunier et al. (2011)	119	Externalizing behavior trajectories: The role of parenting, sibling relationships and child personality	Journal of Applied Developmental Psychology	21.85%	3.9	Europe	SIB
Howe et al. (2011)	40	Sibling Relationship Quality in Early Adolescence: Child and Maternal Perceptions and Daily Interactions	Infant and Child Development	45%	11.5	America	SRQ
Dunn et al. (1999)	3681	Siblings, Parents, and Partners: Family Relationships within a Longitudinal Community Study	The Journal of Child Psychology and Psychiatry and Allied Disciplines	48.3%	7.3	Europe	SRI
Zheng et al. (2020)	788	The Relationship Between non-only-children High Grade Pupils’ Parenting Styles and Sibling Relationship in The two-child Policy Context: The Mediating Effect of Theory of Mind	Psychological Exploration	52.03%	11.32	Asia	S1RQ
Du (2020)	181	Impact of parential rearing patterns on sibling relationships in firstborn children	Master Degree Thesis of Central China Normal University	38.12%	4.99	Asia	PEPC-SRQ
Li (2019)	109	Firstborn Children’s Jealousy and Capability of Emotion Regulation in Sibling Relationships: The Regulating Effect of Fathering Pattern	Master Degree Thesis of China West Normal University	55.96%	9.79	Asia	Other
Yang (2020)	882	The characteristics and related factors of emotional and behavioral problems of preschool firstborn children during transition to siblinghood in Chongqing	Master Degree Thesis of Chongqing Medical University	54.89%	3.58	Asia	Other
Han (2020)	1365	Research on Sibling Relationship of First-born Young Children in Two-Child Families	Master Degree Thesis of Northeast Normal University	61.70%	5.16	Asia	SRQ
Su et al. (2022)	649	The influence of parenting style on sibling relationship among young children	The Family Education of China	46.40%	4.37	Asia	PEPC-SRQ
Wang (2020)	1493	Adolescent sibling violence: the influence of family factors	Master Degree Thesis of Hunan Agricultural University	49.97%	12.84	Asia	SBQ
